# Pathogenic characteristics of a QX-like infectious bronchitis virus strain SD in chickens exposed at different ages and protective efficacy of combining live homologous and heterologous vaccination

**DOI:** 10.1186/s13567-020-00811-y

**Published:** 2020-07-08

**Authors:** Lei Shao, Jing Zhao, Lihua Li, Xiuying Huang, Huiming Yang, Jinlong Cheng, Changqing Liu, Guozhong Zhang

**Affiliations:** 1 Key Laboratory of Animal Epidemiology of the Ministry of Agriculture, College of Veterinary Medicine, China Agricultural University, No. 2 Yuanmingyuan West Road, Haidian, Beijing, 100193 China; 2 Beijing Huadu Yukou Poultry Company Limited, Beijing, 101206 China

**Keywords:** Infections bronchitis virus, Pathogenicity, Chicken, Vaccine, Efficacy

## Abstract

Continued reports of infections with infectious bronchitis virus (IBV) variants have occurred since its first isolation in the 1930s. Currently, QX-like IBVs are the predominant circulating genotype around the world. Here, the pathogenicity of QX-like IBV strain SD was characterized in chickens at different ages of exposure to the virus, and the protection efficacy of available vaccine combinations against IBV was evaluated. The results revealed that QX-like IBV strain SD was severely pathogenic in chickens, causing respiratory, urinary and reproductive infections, irrespective of age, based on clinical observations, viral distribution in tissues and a ciliostasis study. Severe respiratory signs, tracheal cilia injury, nephritis and abnormal development of the oviduct and ovarian follicles were evident throughout the experiment. A challenge experiment demonstrated that the homologous QX vaccine showed superior protection efficacy compared with other available vaccines, confirming the importance of IBV vaccine seed homology against the circulating IBV strains. Our findings aid an understanding of the pathogenicity of QX-like IBVs that may help to further control the infection.

## Introduction

Coronaviruses (CoVs) are enveloped viruses with a positive sense, single-stranded RNA genome that can be divided into four groups: α-CoVs, β-CoVs, γ-CoVs and δ-CoVs based on genetic and antigenic criteria [1]. CoVs primarily cause enzootic infections in birds, animals and humans [2–4]. However, at the beginning of the 21st century, CoVs emerged that are highly transmissible and pathogenic to humans, including severe acute respiratory syndrome coronavirus (SARS-CoV), Middle East respiratory syndrome coronavirus (MERS-CoV) and a novel coronavirus (2019-nCoV) [5–7].

Infectious bronchitis virus (IBV) belongs to the γ-CoVs that cause avian infectious bronchitis (IB), a highly contagious disease of chickens [8]. IB occurs globally, both in chickens raised on a large and a small scale, leading to significant economic losses to the poultry industry each year [9, 10]. Like other positive-sense single-stranded RNA viruses, IBV has a high mutation rate that results in the continuous emergence of new serotypes and variants [11–14]. IBV exists as numerous serotypes, which causes difficulties with regard to the prevention and control of the disease [15–17]. IB therefore remains a serious threat to chicken production in many countries around the world [18–22].

Among the numerous genotypes, QX-like IBVs have emerged and gradually evolved into the predominant circulating genotype worldwide since the 1990s [18, 23–26]. Analyses of genetic evolution, antigenicity and pathogenicity reveal many major differences among QX-like IBVs and other serotypes [17, 27–29]. Several QX-like IBV vaccines have also been developed to combat infections induced by the virus [30–33].

In this study, the pathogenic characteristics of QX-like IBV strain SD in 2-, 3-, 8-, 18- and 22-week-old specific-pathogen-free (SPF) chickens were characterized. The efficacy of a combination of vaccines based on Massachusetts (Ma5 and H120) and variant (LDT3-A and QX-like SZ160) strains against QX-like IBV strain SD was also assessed. Our findings offer new insight into the pathogenicity of QX-like IBVs in chickens exposed at different ages, and may aid the development of novel infection control strategies.

## Materials and methods

### Virus strain and virus titration

The QX-like IBV strain SD (GenBank accession no.: KY421673) was isolated from chicken flocks vaccinated with Mass-type IBV vaccines in 2013 that showed obvious respiratory symptoms and renal disease [27]. The virus was propagated by inoculating 10-day-old SPF embryonated chicken eggs via the allantoic cavity route. The 50% embryo infectious dose (EID_50_) was determined as described [34].

### Animals and ethics statement

All SPF embryonated chicken eggs and SPF chickens used in this study were purchased from Beijing Boehringer Ingelheim Vital Biotechnology Co. Ltd. (Beijing, China). The protocols and procedures used in these experiments, including the possibility of animal death without euthanasia, were specifically considered and approved by the Animal Welfare and Ethical Censor Committee at China Agricultural University (CAU approval number 2020-08).

### Experimental design for pathogenicity

To evaluate the pathogenicity of QX-like IBV strain SD in chickens at different ages of exposure to the virus, a total of 162 SPF chickens were divided into six groups. Chickens in groups 1 (n = 30), 2 (n = 30), 3 (n = 22), 4 (n = 15) and 5 (n = 15) were inoculated via the oculo-nasal route with 200 µl of medium containing 10^6.0^ EID_50_ of IBV strain SD at the ages of 2-, 3-, 8-, 18- and 22-weeks old, respectively. In each virus-infected age, another 10 chickens were not challenged as controls (group 6).

The birds were monitored daily for clinical signs. Each group was sampled at 5, 7, 9 and 14 days post-inoculation (dpi) to measure the ciliary activity in the trachea, and to observe the gross lesions in the larynx trachea, kidney, oviduct and ovarian follicle, and to detect the level of virus RNA in the trachea, kidney, bursa, proventriculus and oviduct by RT-PCR. Two birds from each group were randomly selected and humanly euthanized by cervical dislocation at each sampling date. The body weight of chickens from each group was measured at 0 and 14 dpi. Blood was also taken for antibody detection at 9–16 dpi, prior to euthanization.

### Experimental design for vaccine efficacy

Two commercial IBV vaccines belong to Mass serotype (Ma5 and H120) and another two local variant vaccines of China belong to GI-19 genotype (LDT3-A and QX-like SZ160) were used in this study. The vaccines were inoculated at the manufacturer’s recommended dose. The experimental design for the immunization and challenge tests is shown in Table 1. Briefly, one hundred 1-day-old Jinghong chickens (Chinese local layer) were divided into 10 groups (10 chicks/group) in separate chicken isolators. Birds in group A–C were vaccinated with Ma5, LDT3-A or QX vaccine at one day of age, respectively. Birds in group D-F were vaccinated with Ma5, LDT3-A or QX vaccine at 1day of age, with a booster with H120 vaccine at 21 days of age. Birds in group G were vaccinated with QX vaccine at 1 day and 21 days of age, respectively. The last three groups were non-vaccinated challenged (group H and I) and non-challenged control (group J). Birds in group A–C and H were challenged via the ocular-nasal route with the QX-like IBV strain SD (10^6.0^ EID_50_/bird) at 21 days post vaccination (21 days of age). Birds D–G and I were challenged via the ocular-nasal route with the QX-like IBV strain SD (10^6.0^ EID_50_/bird) at 14 days post vaccination after the booster vaccination (34 days of age). After 7 days post-challenge (dpc), three birds from each group were necropsied to observe gross lesions and tracheal samples were collected and examined by ciliostasis tests.

**Table 1:** Experimental design of the immunization and challenge tests against QX-like IBV strain SD in chickens^a^.

Group	No. of chickens	Age (d)	Vaccine	Route	Challenge		Route and dose of challenge
					Day 21	Day 35	
A	10	1	Ma5^b^	Oculo-nasally	IBV strain SD	–	Oculo-nasally, 10^6^ EID_50_/bird, 0.2 mL
B	10	1	LDT3-A^b^	Oculo-nasally	IBV strain SD	–	Oculo-nasally, 10^6^ EID_50_/bird, 0.2 mL
C	10	1	QX^b^	Oculo-nasally	IBV strain SD	–	Oculo-nasally, 10^6^ EID_50_/bird, 0.2 mL
D	10	1	Ma5 + H120^c^	Oculo-nasally	–	–	Oculo-nasally, 10^6^ EID_50_/bird, 0.2 mL
E	10	1	LDT3-A + H120^c^	Oculo-nasally	–	IBV strain SD	Oculo-nasally, 10^6^ EID_50_/bird, 0.2 mL
F	10	1	QX + H120^c^	Oculo-nasally	–	IBV strain SD	Oculo-nasally, 10^6^ EID_50_/bird, 0.2 mL
G	10	1	QX + QX^d^	Oculo-nasally	–	IBV strain SD	Oculo-nasally, 10^6^ EID_50_/bird, 0.2 mL
H	10	1	PBS	Oculo-nasally	IBV strain SD	–	Oculo-nasally, 10^6^ EID_50_/bird, 0.2 mL
I	10	1	PBS + PBS	Oculo-nasally	–	IBV strain SD	Oculo-nasally, 10^6^ EID_50_/bird, 0.2 mL
J	10	1	–	–	–	–	Oculo-nasally, PBS, 0.2 mL

^a^All chickens are local egg-producing chickens.

^b^Vaccination at day 1.

^c^Ma5, LDT3-A or QX vaccination at day 1, H120 vaccination at day 21.

^d^First vaccination at day 1, second at day 21.

### Inhibition of ciliary activity

To evaluate tracheal ciliostasis, three sections of the upper, middle and lower part of the trachea (i.e., nine rings per bird) were analysed and the average ciliostasis score was calculated as previously described [35]. A score of 0 was given if the cilia in the complete tracheal section showed movement; a score of 1 was given if the cilia of 75%–100% of the tracheal section showed movement; a score of 2 if the cilia of 50%–75% of the trachea showed movement; 3 if the cilia of 25%–50% of the trachea section showed movement; and 4 if the cilia of less than 25% of the trachea section showed movement or no movement at all.

### IBV detection by reverse transcription polymerase chain reaction (RT-PCR)

The total RNA of the tissues (trachea, kidney, bursa, proventriculus and the middle part of oviduct) was extracted using Trizol reagent (Invitrogen, Carlsbad, CA, USA) according to the manufacturer’s instructions. Reverse transcription was conducted at 37 °C for 1 h, with 3 μg of total RNA, 1 μL of random hexamer primers (500 μg/mL) (Promega, Madison, WI, USA) and 0.5 μL of M-MLV reverse transcriptase (200 U/μL) (Promega). PCR was performed at 94 °C for 3 min, followed by 30 cycles of denaturation (94 °C, 40 s), annealing (53 °C, 30 s) and polymerization (72 °C, 45 s), and the postpolymerization step was performed at 72 °C for 7 min. Amplified sequences were analysed by 1.2% agarose gel electrophoresis. PCR was performed using a pair of primers (forward: 5′- GGAAGATAGGCATGTAGCTT -3′; reverse: 5′- CTAACTCTATACTAGCCTAT -3′), which amplify and detect a 740-bp fragment of the S1 gene of IBV.

### Antibody response

To detect IBV antibodies, serum samples were tested with a commercial IBV enzyme-linked immunosorbent assay (ELISA) kit (BioChek, Reeuwijk, The Netherlands) according to the manufacturer’s instructions. Sera with sample/positive (S/P) ratios above the cut-off level of 0.20 (titre ≥ 834) were considered positive.

### Statistical analysis

Ciliary activity and antibody level were statistically evaluated by one-way or two-way ANOVA tests adjusted for post hoc analysis, followed by Bonferroni’s multiple comparison tests. For all tests, the following notations are used to indicate significant differences between groups: *p < 0.05; **p < 0.01; ***p < 0.001. All data were analysed using GraphPad Prism 6.01 (GraphPad, San Diego, CA, USA).

## Results

### Clinical signs in chickens at different ages of exposure to the virus

**Figure 1:**
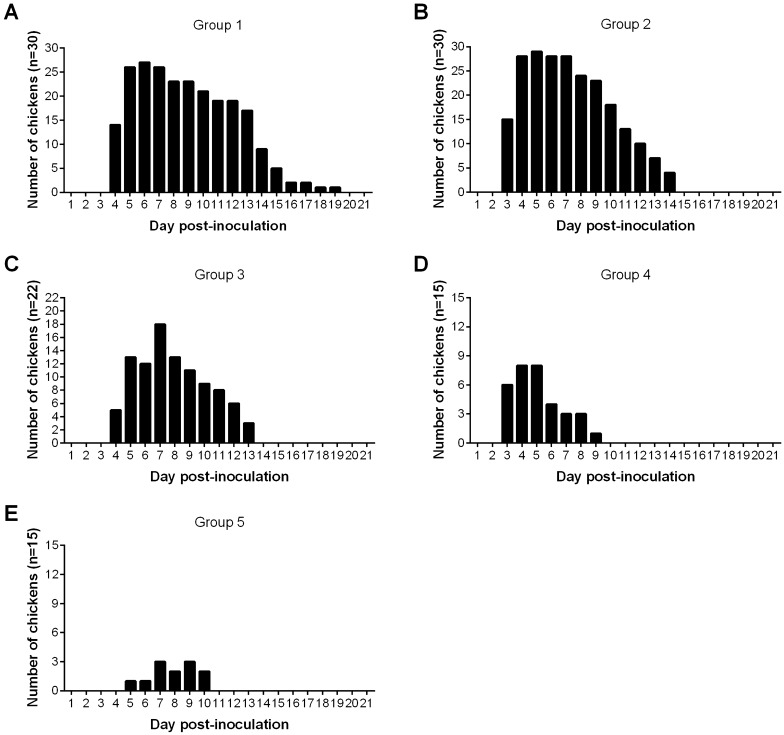
**Proportion and duration of clinical signs in chickens of different groups after infection with IBV strain SD. A** Group 1, chickens were infected with 10^6.0^ EID_50_ of IBV strain SD at 2-weeks old. **B** Group 2, chickens were infected with 10^6.0^ EID_50_ of IBV strain SD at 3-weeks old. **C** Group 3, chickens were infected with 10^6.0^ EID_50_ of IBV strain SD at 8-weeks old. **D** Group 4, chickens were infected with 10^6.0^ EID_50_ of IBV strain SD at 18-weeks old. **E** Group 5, chickens were infected with 10^6.0^ EID_50_ of IBV strain SD at 22-weeks old.

Chickens infected at different ages with QX-like IBV strain SD showed obvious clinical signs, whereas the chickens in the control group remained healthy throughout the study. The most prominent clinical signs were characterized by depression, sneezing, nasal discharge, tracheal rales and difficulty in breathing, and respiratory clinical signs were predominant in all of the inoculated groups (Table 2). Clinical monitoring of infected chicks revealed that apparent symptoms began at 3 dpi. However, the incidence rate and duration of the clinical signs was different between the groups (Figure  1). The clinical signs in infected chickens of younger age (groups 1 and 2) were more severe than in older chickens (groups 3, 4 and 5). The mortality rates in virus-infected groups 1 to 5 were 13.3%, 6.7%, 13.6%, 13.3% and 6.7%, respectively.

**Table 2:** Summarized clinical signs of SPF chickens infected with 10^6.0^ EID_50_ of QX-like IBV strain SD at different ages.

Group	Clinical signs							
	No Signs	Diarrhea	Sneezing	Nasal discharge	Tracheal rale	Dyspnea	Depression	Dead
1	1/30^a^	17/30	26/30	26/30	13/30	23/30	26/30	4/30
2	0/30	15/30	24/30	29/30	16/30	27/30	22/30	2/30
3	0/22	1/22	4/22	17/22	7/22	2/22	8/22	3/22
4	4/15	0/15	3/15	3/15	7/15	7/15	4/15	2/15
5	8/15	0/15	0/15	4/15	0/15	1/15	3/15	1/15
6	50/50	0/50	0/50	0/50	0/50	0/50	0/50	0/50

Group 1, 2-weeks-old chickens infected with IBV strain SD. Group 2, 3-weeks-old chickens infected with IBV strain SD. Group 3, 8-weeks-old chickens infected with IBV strain SD. Group 4, 18-weeks-old chickens infected with IBV strain SD. Group 5, 22-weeks-old chickens infected with IBV strain SD. Group 6, control group.

^a^Number of chickens exhibiting clinical signs out of the total number of birds.

### Gross lesions in chickens at different ages of exposure to the virus

**Figure 2:**
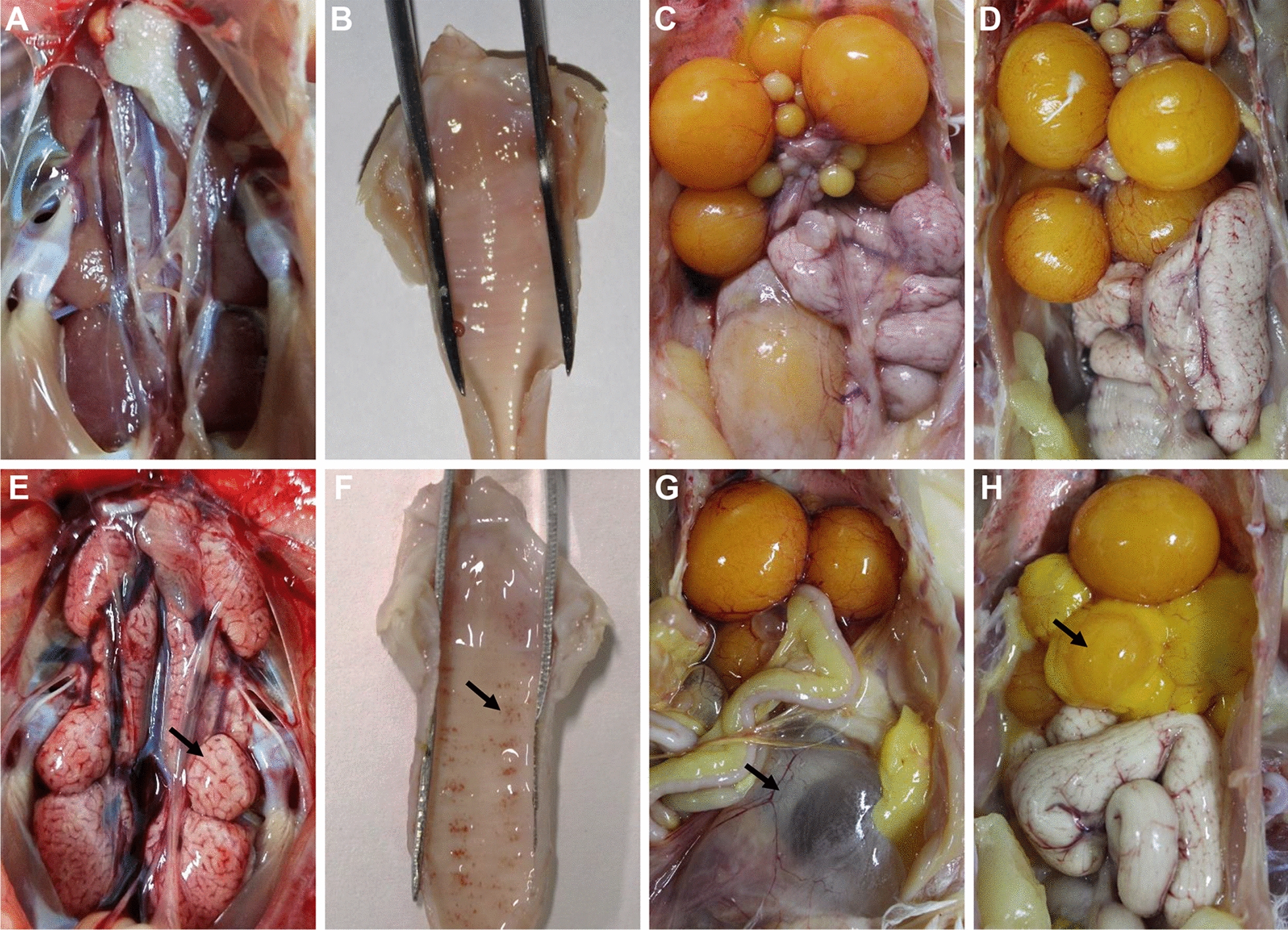
**Characteristic lesions in the different organs of chickens after infection with IBV strain SD at different ages. A–D** kidney, larynx trachea, oviduct and ovarian follicle of controls, respectively. **E** kidneys were swollen with urate deposits. **F** punctate haemorrhages and catarrhal exudates in the larynx and trachea. **G** dilatation and suffusion of the oviduct. **H** deformation and atrophy of the ovarian follicles. Black arrows indicate lesions detected in the tissue.

Similar gross lesions in the larynx, trachea, kidney, oviduct and ovarian follicle were observed in the different groups inoculated with QX-like IBV strain SD (Table 3). These gross lesions included catarrhal exudate and haemorrhages in the larynx or trachea, renal enlargement and urate with varying severity, and abnormal development of the oviduct and ovarian follicle (Figure  2). Haemorrhagic tracheitis and nephrosis-nephritis with varying severity were observed occasionally during the whole experiment. The characteristic dilatation in different part of the oviduct and abnormal development of the ovarian follicle were also observed in all inoculated groups. These results revealed that QX-like IBV strain SD can induce disease in the respiratory system, urinary system and reproductive system simultaneously.

**Table 3:** Gross lesions in SPF chickens infected with QX-like IBV strain SD at different ages.

Group	Lesions	Days post inoculation (dpi)				
		5	7	9	14	X^a^
1	Tracheal exudate/hemorrhage	0/2^b^	2/2	2/2	0/2	–
	Laryngeal exudate/hemorrhage	2/2	2/2	0/2	0/2	–
	Renal enlargement/urate	1/2	2/2	1/2	2/2	–
	Abnormal follicular development	–	–	–	–	6/18
	Abnormal development of oviduct	–	–	–	–	9/18
2	Tracheal exudate/hemorrhage	0/2	1/2	1/2	2/2	–
	Laryngeal exudate/hemorrhage	0/2	0/2	0/2	2/2	–
	Renal enlargement/urate	2/2	1/2	1/2	2/2	–
	Abnormal follicular development	–	–	–	–	8/20
	Abnormal development of oviduct	–	–	–	–	3/20
3	Tracheal exudate/hemorrhage	2/2	2/2	1/2	2/2	–
	Laryngeal exudate/hemorrhage	1/2	0/2	2/2	2/2	–
	Renal enlargement/urate	0/2	2/2	2/2	2/2	–
	Abnormal follicular development	–	–	–	–	1/11
	Abnormal development of oviduct	–	–	–	–	1/11
4	Tracheal exudate/hemorrhage	2/2	1/2	2/2	2/2	–
	Laryngeal exudate/hemorrhage	1/2	1/2	0/2	0/2	–
	Renal enlargement/urate	1/2	0/2	1/2	2/2	–
	Abnormal follicular development	–	–	–	–	2/7
	Abnormal development of oviduct	–	–	–	–	1/7
5	Tracheal exudate/hemorrhage	2/2	2/2	0/2	–	–
	Laryngeal exudate/hemorrhage	0/2	0/2	0/2	–	–
	Renal enlargement/urate	1/2	2/2	2/2	–	–
	Abnormal follicular development	–	–	–	–	3/9
	Abnormal development of oviduct	–	–	–	–	1/9
6	Tracheal exudate/hemorrhage	0/10	0/10	0/10	0/8	–
	Laryngeal exudate/hemorrhage	0/10	0/10	0/10	0/8	–
	Renal enlargement/urate	0/10	0/10	0/10	0/8	–
	Abnormal follicular development	–	–	–	–	0/12
	Abnormal development of oviduct	–	–	–	–	0/12

Group 1, 2-weeks-old chickens infected with IBV strain SD. Group 2, 3-weeks-old chickens infected with IBV strain SD. Group 3, 8-weeks-old chickens infected with IBV strain SD. Group 4, 18-weeks-old chickens infected with IBV strain SD. Group 5, 22-weeks-old chickens infected with IBV strain SD. Group 6, control group.

^a^End of observation period at 168 d.

^b^Number of chickens exhibiting gross lesions out of the examined number of birds.

### Tracheal ciliary activity in chickens at different ages of exposure to the virus

**Figure 3:**
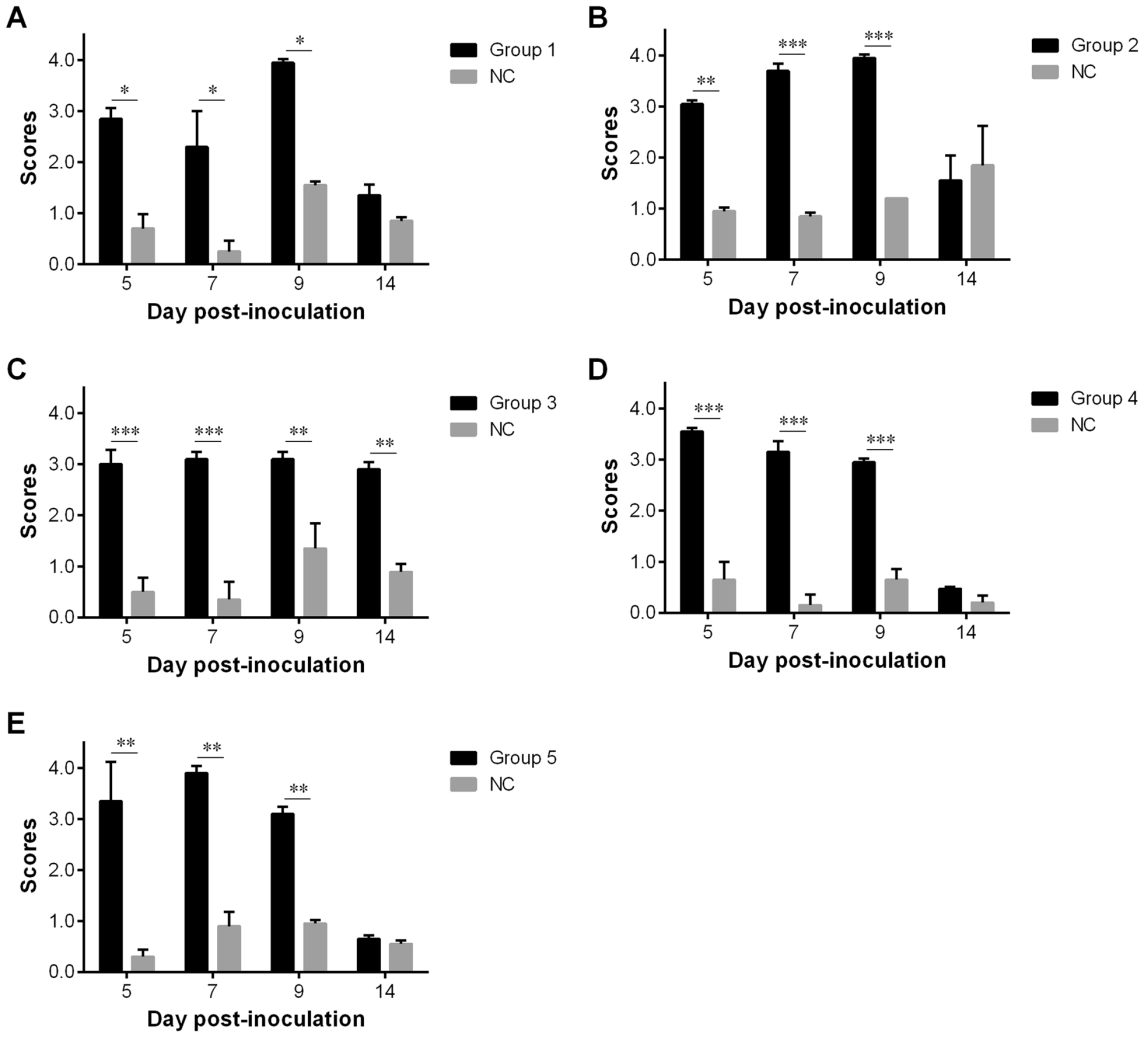
**Mean scores in the ciliary activity test at 5, 7, 9 and 14** **days post-inoculation (dpi) after infection with IBV strain SD. A** Group 1, chickens were infected with 10^6.0^ EID_50_ of IBV strain SD at 2-weeks old. **B** Group 2, chickens were infected with 10^6.0^ EID_50_ of IBV strain SD at 3-weeks old. **C** Group 3, chickens were infected with 10^6.0^ EID_50_ of IBV strain SD at 8-weeks old. **D** Group 4, chickens were infected with 10^6.0^ EID_50_ of IBV strain SD at 18-weeks old. **E** Group 5, chickens were infected with 10^6.0^ EID_50_ of IBV strain SD at 22-weeks old. *NC* in each panel, not challenged. Statistical significance was considered as follows: significant at p < 0.05 (*), highly significant at p < 0.01 (**) and very highly significant at p < 0.001 (***).

Tracheal ciliary activity was observed from all groups at 5, 7, 9 and 14 dpi. The severity of the ciliostasis in chickens at different ages of exposure to the QX-like IBV strain SD proved to be similar, sometimes leading to severe ciliostasis, compared with the control group (p < 0.05, 0.01 or 0.001). The highest inhibitory activity was observed between 5 and 9 dpi, while partial recovery of ciliary activity was observed at 14 dpi in the infected chickens, with the exception of group 3 (Figure  3).

### Virus distribution in chickens at different ages of exposure to the virus

**Figure 4:**
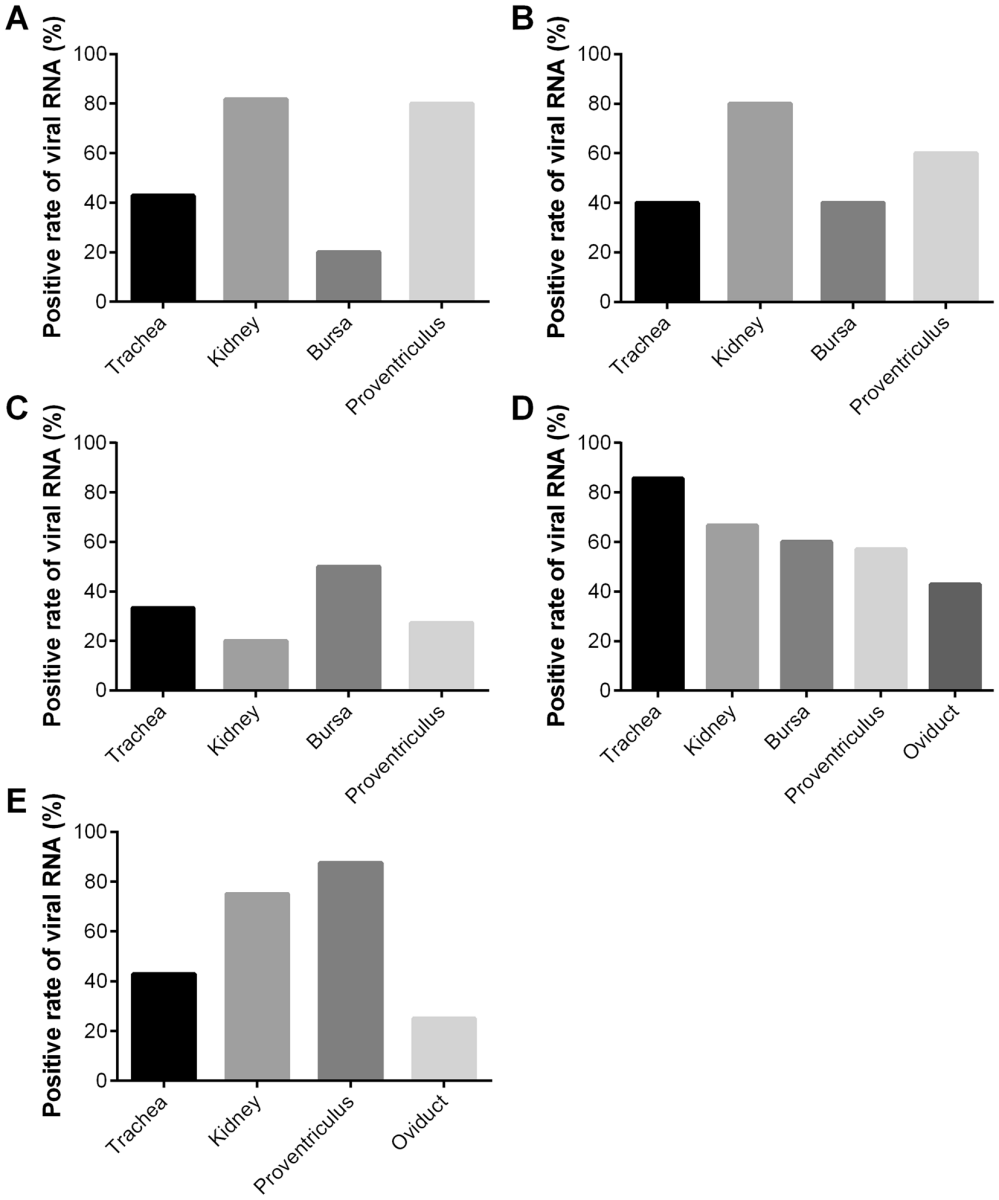
**Detection of viral RNA by RT-PCR in the trachea, kidney, bursa, proventriculus and oviduct. A–E** The chickens were infected with 10^6.0^ EID_50_ of IBV strain SD at 2-, 3-, 8-, 1-8 and 22-weeks old, respectively. Viral RNA % indicate the number of positive/total samples.

The presence of the virus was detected in all sampled tissues at different dpi by the RT-PCR test (Figure  4A–E). No virus was detected in any tissues of the chickens in control group. The viral antigens were found in all sampled tissues, including the trachea, kidney, bursa, proventriculus and oviduct. However, the proportion of positive samples varied in the different organs between the groups. In the trachea, the highest positive rate for IBV RNA was 85.7% in group 4. In the kidney, the positive rates were 81.8%, 80%, 20%, 66.7% and 75%, respectively. In the proventriculus, the positive rates varied from 27.3% to 87.5%. In the bursa and oviduct, the average positive rates were 42.5% and 34.0%, respectively. Among all of the tested tissues, viral RNA in the kidney had the highest positivity of 64.70% compared with other tissues, followed by the proventriculus at 62.4%.

### Average body weight of chickens at different ages of exposure to the virus

**Figure 5:**
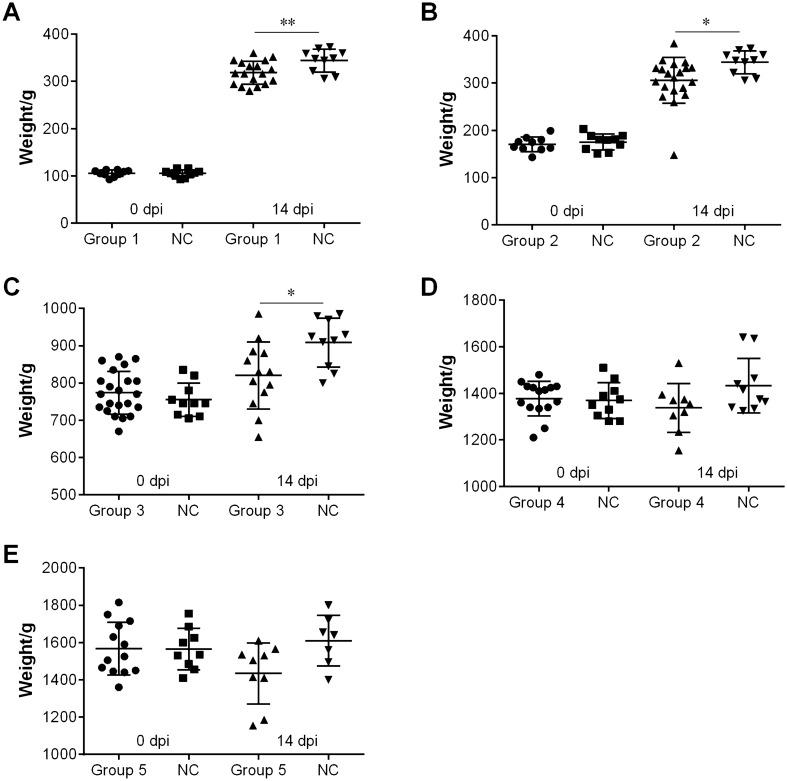
**Body weight of birds from the different groups at 0 and 14** **days post-inoculation (dpi). A** Group 1, chickens were infected with 10^6.0^ EID_50_ of IBV strain SD at 2-weeks old. **B** Group 2, chickens were infected with 10^6.0^ EID_50_ of IBV strain SD at 3-weeks old. **C** Group 3, chickens were infected with 10^6.0^ EID_50_ of IBV strain SD at 8-weeks old. **D** Group 4, chickens were infected with 10^6.0^ EID_50_ of IBV strain SD at 18-weeks old. **E** Group 5, chickens were infected with 10^6.0^ EID_50_ of IBV strain SD at 22-weeks old. *NC* in the different panels, not challenged. Statistical significance was considered as follows: significant at p < 0.05 (*) and highly significant at p < 0.01 (**).

Body weight was measured in the different groups inoculated with QX-like IBV strain SD at 0 and 14 dpi (Figure  5). The average weight of infected chickens belonging to the younger age groups (1, 2 and 3) was significantly lighter compared with the control group (p < 0.05 or 0.01).

### Serological response of chickens at different ages of exposure to the virus

**Figure 6:**
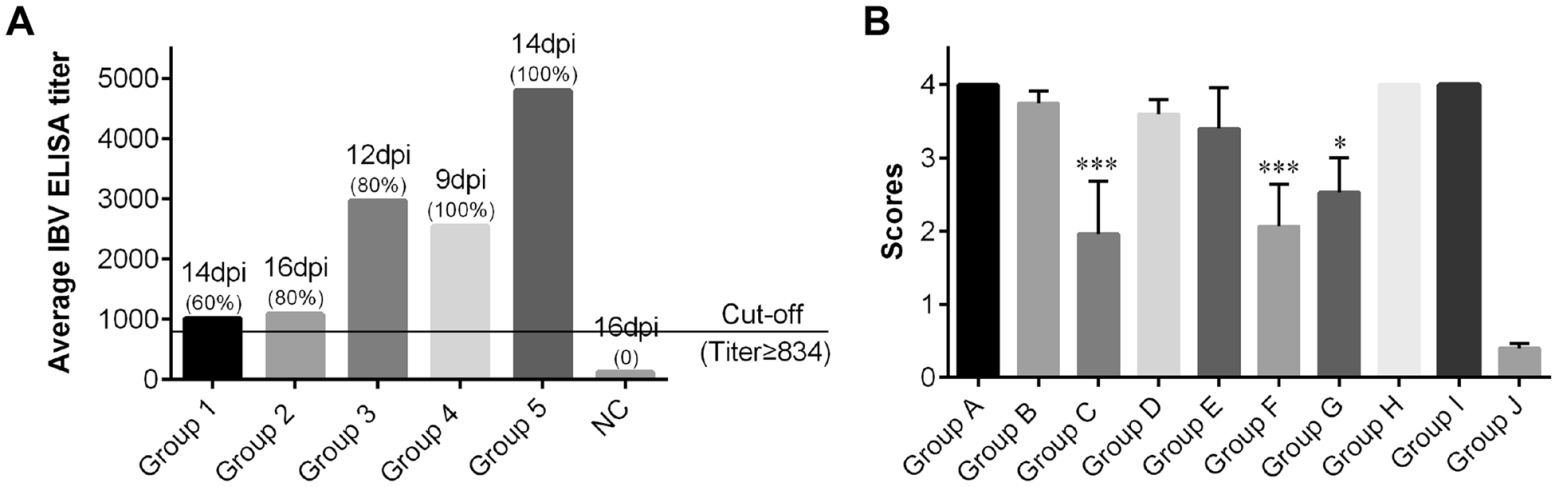
**Antibody response in pathogenicity test and ciliostasis scores in vaccine efficacy test**. **A** Antibody response induced by IBV strain SD at 9–16 days post-inoculation (dpi) in chickens inoculated at different ages. Serum with a titre ≥ 834 was considered positive. The numbers in bracket indicate the positive rate for IBV antibody in different groups. *NC*, not challenged. **B** ciliostasis scores in the vaccination groups at 7 days post-challenge (dpc) with IBV strain SD.

The collected sera from chickens of the different groups were measured for antibody levels against IBV using an ELISA kit (BioChek) (Figure  6A). In all of the inoculated groups, serum samples were negative for IBV antibody at day 0 but the mean antibody titres induced by QX-like IBV strain SD were increased at 9–16 dpi, with a positive rate of 60%, 80%, 80%, 100% and 100% from group 1 to group 5, respectively. The serum of the control chickens was free from specific antibodies against IBV throughout the study.

### Protective efficacy of the different vaccinations against IBV strain SD challenge

The clinical signs, gross lesions and mortality rates of all groups are summarized in Table 4. Chickens in the non-vaccinated group challenged with QX-like IBV strain SD showed obvious clinical manifestations including depression, respiratory symptoms (sneezing, tracheal rales and difficulty breathing), catarrhal exudate in the trachea, renal enlargement and urate deposition, with a mortality rate of 40% among the chickens infected at 21 d. Some chickens showed depression or respiratory symptoms after a single vaccination with vaccine Ma5 or LDT3-A, and one chicken died in the Ma5 vaccination group. No clinical changes were observed following a single vaccination with vaccine QX. The birds that had been vaccinated with Ma5, LDT3-A or QX and subsequently with the H120 vaccine showed different gross lesions between the groups. The severity of clinical manifestations could be categorized as follows: group D (Ma5 + H120) > group E (LDT3-A + H120) > group F (QX + H120), and group G (QX + QX) was similar to group F.

**Table 4:** Vaccination schedules, clinical signs and gross lesions of vaccinated chickens after challenge with QX-like IBV strain SD.

Groups	Vaccination regime		Clinical signs			Gross lesions	
	Prime1st day	Booster 21th day	Dead	Lethargic and reluctant	Respiratory symptoms	Renal enlargement/urate	Tracheal exudate/hemorrhage
A	Ma5	/^a^	1/9	2/9^b^	5/9	2/9	0/9
B	LDT3-A	/	0/10	2/10	1/10	0/10	0/10
C	QX	/	0/10	0/10	0/10	0/10	0/10
D	Ma5	H120	0/10	0/10	8/10	0/10	2/10
E	LDT3-A	H120	0/10	0/10	4/10	1/10	2/10
F	QX	H120	0/10	0/10	0/10	2/10	1/10
G	QX	QX	0/10	0/10	2/10	1/10	2/10
H	No vaccine challenge (day 21)		4/10	7/10	4/10	4/10	2/10
I	No vaccine challenge (day 35)		0/10	0/10	9/10	0/10	5/10
J	No vaccine No challenge		0/10	0/10	0/10	0/10	0/10

^a^Not performed.

^b^One bird died before challenge.

The QX-like IBV strain SD caused 100% damage to the cilia in the non-vaccinated group at 7 dpc. The immune groups administered a single vaccination with vaccine Ma5 or LDT3-A that were also primed with Ma5 or LDT3-A and boosted with H120, also showed higher ciliostasis scores. However, the average ciliostasis scores in the groups immunized with a single QX vaccine or primed with QX vaccine and boosted with H120 or QX vaccine, were significantly lower compared with other groups and with the control group (p < 0.05 or 0.001) (Figure  6B).

## Discussion

Vaccine immunization is one of the critical measures needed in the prevention of avian IB. It is difficult to control this disease because of the continuous emergence of new IBV variants, resulting in different genotypes, serotypes and pathotypes [9, 36]. It is therefore important to determine the prevalent genotypes or serotypes, and the cross-protective potential of available vaccines and vaccination strategies. QX-like IBVs are currently the predominant genotype worldwide and exhibit severe pathogenicity compared with other strains [17, 26, 37, 38]. In this study, the pathogenicity of QX-like IBV strain SD in chickens of different ages was characterized, and the protection efficacy of some vaccine combinations against the virus was assessed.

The respiratory pathogenicity of QX-like IBV strain SD induced in susceptible chickens was observed in all inoculation groups of different ages, and included sneezing, tracheal rales and difficulty breathing, which indicated injury to the respiratory system. This result was further confirmed by the ciliostasis scores, with QX-like IBV strain SD causing almost 100% damage to the cilia in all of the infected groups of different ages, between 5 and 9 dpi. It is believed that secondary infection by other pathogens, such as *Mycoplasma galliscepticum*, *Escherichia coli* and avian influenza H9N2 virus, would be more likely to occur when the integrity of the respiratory mucosa is compromised [39, 40].

Despite the QX strain being initially isolated from a case of proventriculitis, similar viruses were recovered from cases associated with a drop in egg production and renal damage in subsequent studies [27, 29, 41, 42]. Severe renal pathogenicity was observed in the different infected groups after challenge with QX-like IBV strain SD at different ages. Typical gross lesions of renal enlargement and urate deposition, present at all stages, are shown in Figure  2E. Consistent with the clinical observations, infection with a nephropathogenic IBV strain can induce high mortality [16, 17, 37].

Serious consequences of IB outbreaks are obviously decreased egg shell quality and production in layers and breeders with reduced fertility, and severe, irreversible damage in the immature ovary and oviduct, especially in the early IBV infection of young pullets [43, 44]. In the present study, we further found that QX-like IBV strain SD could cause gross lesions in the reproductive system, including undeveloped oviducts, fluid accumulation in the oviducts, and undeveloped or abnormal development of the ovarian follicle. These results indicated that infection with QX-like IBVs at any stage could damage the reproductive system of chickens, resulting in poor production performance.

A homologous strain can induce the best cross-protection against IBV infection of field strains [32, 35]. The current study further confirmed that QX vaccine had the highest protection efficacy against QX-like IBV strain SD compared with other available vaccines by clinical observations and ciliary activity scores. Despite inducing a certain degree of ciliostasis and viral recombination, live attenuated IB vaccines are critical in IB control [22, 45]. The effect of IBV attenuated vaccines on tracheal ciliary activity deserves attention in poultry production. Taken together, cross-protection studies to determine the appropriate vaccine strains and proper immunization procedures are essential for effective IB control.

In conclusion, this study showed that QX-like IBV strains induce severe pathogenicity in chickens. The viruses are able to simultaneously infect the respiratory system, urinary system and reproductive system of chickens at different ages, which results in poor production performance. To combat QX-like IBV infections, it is critical to develop the appropriate matching vaccine strains and determine the most appropriate vaccination regime.

## Data Availability

All data generated during this study are included in this published article.
